# Convergence of retrotransposons in oomycetes and plants

**DOI:** 10.1186/s13100-017-0087-y

**Published:** 2017-03-14

**Authors:** Kirill Ustyantsev, Alexandr Blinov, Georgy Smyshlyaev

**Affiliations:** 1 Institute of Cytology and Genetics, Laboratory of Molecular Genetic Systems, Prospekt Lavrentyeva 10, 630090 Novosibirsk, Russia; 2 Structural and Computational Biology Unit, European Molecular Biology Laboratory, 69117 Heidelberg, Germany

**Keywords:** Convergent evolution, Retrotransposons, Plants, Oomycetes, Ribonuclease H, Chromodomain

## Abstract

**Background:**

Retrotransposons comprise a ubiquitous and abundant class of eukaryotic transposable elements. All members of this class rely on reverse transcriptase activity to produce a DNA copy of the element from the RNA template. However, other activities of the retrotransposon-encoded polyprotein may differ between diverse retrotransposons. The polyprotein domains corresponding to each of these activities may have their own evolutionary history independent from that of the reverse transcriptase, thus underlying the modular view on the evolution of retrotransposons. Furthermore, some transposable elements can independently evolve similar domain architectures by acquiring functionally similar but phylogenetically distinct modules. This convergent evolution of retrotransposons may ultimately suggest similar regulatory pathways underlying the lifecycle of the elements.

**Results:**

Here, we provide new examples of the convergent evolution of retrotransposons of species from two unrelated taxa: green plants and parasitic protozoan oomycetes. In the present study we first analyzed the available genomic sequences of oomycete species and characterized two groups of Ty3/Gypsy long terminal repeat retrotransposons, namely Chronos and Archon, and a subgroup of L1 non-long terminal repeat retrotransposons. The results demonstrated that the retroelements from these three groups each have independently acquired plant-related ribonuclease H domains. This process closely resembles the evolution of retrotransposons in the genomes of green plants. In addition, we showed that Chronos elements captured a chromodomain, mimicking the process of chromodomain acquisition by Chromoviruses, another group of Ty3/Gypsy retrotransposons of plants, fungi, and vertebrates.

**Conclusions:**

Repeated and strikingly similar acquisitions of ribonuclease H domains and chromodomains by different retrotransposon groups from unrelated taxa indicate similar selection pressure acting on these elements. Thus, there are some major trends in the evolution of the structural composition of retrotransposons, and characterizing these trends may enhance the current understanding of the retrotransposon life cycle.

**Electronic supplementary material:**

The online version of this article (doi:10.1186/s13100-017-0087-y) contains supplementary material, which is available to authorized users.

## Background

Retrotransposons are “copy-and-paste” mobile elements transferred via an RNA intermediate through the process of reverse transcription. Generally, retrotransposons are further subdivided in two major groups: long terminal repeat retrotransposons (LTR-RTs), with their viral descendants (retroviruses), and non-LTR retrotransposons (non-LTR-RTs). The only general structural feature shared between autonomous elements from both groups is the reverse transcriptase (RT) domain, a key enzyme responsible for reverse transcription. In contrast, the set of other encoded activities could largely vary and rely on the life cycle organization and insertion strategy of the retrotransposon [1–3]. Each of these additional domains can have an evolutionary history independent from that of the RT domain. There are multiple examples of independent acquisitions of domains with the same enzymatic activity by the diverse retrotransposons, suggesting the importance of the domain-encoded function for the performance of each element [4–10]. One of these examples is the ribonuclease H (RNH) domain, which has been captured by diverse retrotransposons on different occasions [4–6, 8, 11–14].

RNH activity is required for the removal of an RNA template from a cDNA/RNA hybrid generated during reverse transcription. Retrotransposons rely on either the host genome-encoded RNH enzyme or encode their own RNH domains [4]. For example, non-LTR-RTs often rely on host genome-encoded RNH activity, as the reverse transcription of these transposons occurs directly in the nucleus where the host cellular RNH enzyme is naturally present [4, 15]. Nevertheless, some non-LTR-RTs encode their own RNH. For example, some non-LTR-RTs of oomycetes and plants have acquired RNH closely related to the Archaea-like RNHs (aRNH). Interestingly, these two groups of non-LTR-RTs independently acquired aRNHs [6, 11]. In case of the LTR-RTs, the presence of the element-encoded RNH is obligatory, as reverse transcription occurs in the cytoplasm where no host-encoded enzyme is available [4]. Accordingly, the RNH domain has been detected in all LTR-RTs, and the evolution of the domains follows that of the RT [5]. However, some retroelements, such as retroviruses, have captured additional RNH domains, resulting in a ‘dual’ RNH [4, 5, 16]. Strikingly similar to retroviruses, the Tat LTR-RTs of green plants have acquired an additional RNH domain, aRNH, indicating structural and functional convergence between plant Tat LTR-RTs and vertebrate retroviruses [5].

In the present study, we mined all aRNH-containing retrotransposons from oomycete genomes and provided new examples of convergence in retrotransposons between plants and oomycetes. We identified and characterized two groups of Ty3/Gypsy LTR-RTs, Chronos and Archon, and a subgroup of L1 non-LTR-RTs in the genomes of oomycetes, which to our knowledge has not previously been described. These retrotransposons captured aRNH in the same manner as plant retrotransposons. In addition, we showed that Chronos LTR-RTs also captured a chromodomain (CHD), resembling the evolution of plant Chromoviruses and Ty1/Copia CoDi-I LTR-RTs from the free-living Stramenopiles *Phaeodactylum tricornutum* [7, 17–19].

## Results

### Diversity of aRNH-containing retrotransposons in oomycete genomes

aRNH is a subgroup of the type I RNH, which also includes Fungi/Metazoa-like RNHs (fmRNH) and LTR-RT RNH. While fmRNHs and aRNHs are characterized by the presence of histidine or arginine residues respectively in the active site, LTR-RTs RNHs lack any conserved residues in that position [4, 16]. aRNHs were originally described in the archaeal genomes and were also identified as cellular genes in the genomes of plants and some bacteria [20]. Furthermore, RNH domains that were found in Ty3/Gypsy Tat LTR-RTs and Ta11 L1 non-LTR-RTs of higher plants [12–14] were shown to be phylogenetically related to cellular-like aRNHs [5, 6]. In addition, Kojima and Jurka [11] identified a subgroup of aRNH-containing non-LTR-RTs of the Utopia group in oomycete genomes.

To determine the presence of the aRNH in other retroelements, we screened for aRNH sequences in Repbase Update (RU, v. 20.08), the database of eukaryotic transposable elements [21, 22]. Consistent with previous data, all retrotransposons predicted to have an aRNH domain (see Methods for details) were detected in either the genomes of higher plants or the parasitic protozoans oomycetes. Surprisingly, in addition to the previously described Utopia non-LTR-RTs [11], some oomycete Ty3/Gypsy LTR-RTs and L1 non-LTR-RTs also encode aRNH (for the RU accession numbers see Additional file 1: Table S1).

**Fig. 1:**
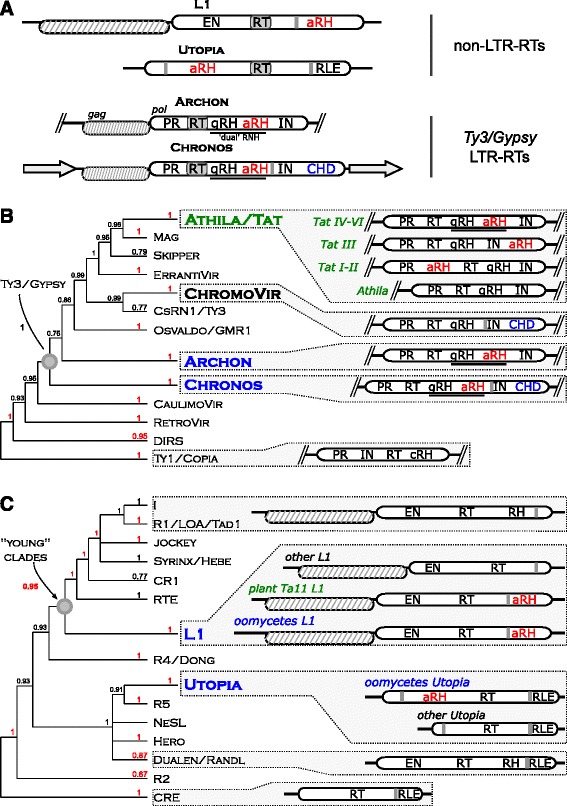
Diversity of aRNH-containing retrotransposons in oomycetes. **a** Schematic structural composition of the elements from the identified groups: ORFs are shown as horizontal ovals (ORFs 1 are shaded); PR – protease; gRH – RNH of Ty3/Gypsy LTR-RTs; aRH – aRNH (in red); IN – integrase; CHD – chromodomain (in blue); EN – apurinic/apyrimidinic endonuclease-like endonuclease, RLE – restriction-like endonuclease; CCHC Zn finger motif indicated as vertical gray line; gray arrows, LTRs – long terminal repeats. **b** Consensus of Maximum-likelihood and Bayesian trees based on the amino acid sequences of RT domain of LTR-RTs. Approximate likelihood-ratio test (aLRT) statistical support values (unit fractions) are shown at the corresponding nodes of the tree; the values are highlighted in red if the corresponding node was additionally supported by more than 60 of 100 bootstrap replicates. Groups of aRNH-containing retrotransposons of oomycetes and plants are emphasized in bold and highlighted in blue and green, respectively. CHD-containing retrotransposons without aRNH of Chromoviruses (ChromoVir) group are emphasized in bold. On the right from the tree schemes of the consensus structures of the ORF2 of the corresponding groups are shown; cRH – RNH of Tc1/Copia LTR-RTs. The complete Maximum-likelihood and Bayesian phylogenetic trees with accession numbers, the names of the elements, and all the statistical support values are presented in Additional file 2: Figure S1. **c** Consensus of Maximum-likelihood and Bayesian trees based on the amino acid sequences of RT domain of non-LTR-RTs. Approximate likelihood-ratio test (aLRT) statistical support values (unit fractions) are shown at the corresponding nodes of the tree; the values are highlighted in red if the corresponding node was additionally supported by more than 60 of 100 bootstrap replicates. On the right of the tree, the schemes of the consensus structures of the corresponding groups are shown; RH – RNH domain of non-LTR-RTs. The complete Maximum-likelihood and Bayesian phylogenetic trees with accession numbers, the names of the elements, and all the statistical support values are presented in Additional file 2: Figure S1

Since the variability of the oomycete retrotransposons annotated and deposited in RU 20.08 was restricted only to retrotransposons from seven species, of which retrotransposons from only four species contained aRNH (Additional file 1: Table S1), to provide comprehensive insight into the diversity of the identified elements, we further analyzed oomycete genomic sequences for the presence of aRNH-containing retrotransposons. This mining resulted in an overall set of 2899 distinct retrotransposon sequences from 21 out of 25 analyzed oomycete genomes. We initially classified the identified elements into the three groups, Ty3/Gypsy, L1 and Utopia, based on homology to the ORF2 amino acid sequences of aRNH-containing retrotransposons identified in RU. When possible, full-length copies were retrieved as representatives for each genome, and their structure and domain composition were analyzed (Fig. 1a, Additional file 1: Table S2).

Based on the RT phylogeny and comparative structural analysis, we identified two groups of aRNH-containing Ty3/Gypsy LTR-RTs in oomycetes. The first group, designated here as Archon, is specific for Saprolegniales genomes, and its members have an aRNH next to the original Ty3/Gypsy RNH domain. Interestingly, this RNH-aRNH junction resembles the ‘dual’ RNH domains of Tat LTR-RTs and retroviruses [5]. The second group, named Chronos, comprises elements detected in the Peronosporales and Pythiales genomes. In addition, a single copy of a Chronos element was identified in *Aphanomyces astaci* (Saprolegniales). These retrotransposons also have ‘dual’ RNH domains. However, in contrast to all other known aRNH-containing elements, these transposons possess a CHD in the 3′ end of their *pol* next to the INT domain (Fig. 1b, Additional file 2: Figure S1, Additional file 1: Table S2). Previously, the presence of a CHD was shown only for two groups of LTR-RTs: Chromoviruses (a group of Ty3/Gypsy LTR-RTs [7, 9, 18, 23]) and CoDi-I elements (a group of Ty1/Copia LTR-RTs from the free-living Stramenopiles, pennate diatom, *Phaeodactylum tricornutum* [17]). Although Archon and Chronos LTR-RTs share similar structural organization with Tat LTR-RTs and Chromoviruses, they seem to be only distantly related to these elements (Fig. 1b, Additional file 2: Figure S1).

Identified in most of the Peronosporales and Pythiales genomes and undetectable in the Saprolegniales genomes (Additional file 1: Table S2), oomycete aRNH-containing L1 elements are similar in general organization to aRNH-containing Ta11 L1 of plants (Fig. 1c). In both groups, the aRNH domain is positioned at the C-terminal end of ORF2. Notably, both groups are also characterized by a CCHC cysteine motif located upstream of the aRNH. In other non-LTR-RTs harboring an RNH, the CCHC is positioned downstream of the RNH in ORF2 [24]. However, despite the similarities in the general organization of ORF2 (Fig. 1c, Additional file 3: Figure S2), oomycete and plant L1s do not form a monophyletic clade within the L1 group.

Oomycete Utopia elements were identified in most Peronosporales and Pythiales genomes, while only one copy was detected in *Saprolegnia diclina* (Saprolegniales) (Additional file 1: Table S2). Utopia is one of the “old” clades of non-LTR-RTs (such as R2, R5, and CRE) and its elements have sequence-specific restriction-like endonuclease domain (RLE), which guides their insertion to U2 small nuclear RNA genes [11]. The Utopia elements identified in our study did not differ in organization from the original Utopias identified by Kojima and Jurka [11] (Fig. 1c, Additional file 3: Figure S2).

The distinct positions of the oomycete Chronos, Archon, L1, and Utopia groups on the RT phylogenetic trees from all previously known aRNH-containing retrotransposons and from each other suggested that aRNH was independently acquired by each of these groups. However, to further elaborate on this idea, we performed a comparative analysis of the aRNHs from genomes of oomycetes, plants and other organisms.

### Diversity of aRNH in oomycetes

After screening the oomycete genomic sequences, we detected aRNHs that were not associated with RT (individual aRNHs) and could therefore represent potential cellular genes. To obtain reference cellular RNH sequences, we additionally screened for fmRNHs using a set of sequences from a previous study [5]. Table 1 summarizes the results of the analysis comparing the distribution of individual aRNHs and fmRNHs to that of the RT-associated aRNH domains. We identified individual aRNHs in 21 out of 25 oomycete genomes. Notably, we previously identified aRNH-containing retrotransposons in these same 21 genomes. In contrast, fmRNH was identified in all studied genomes. For a majority of the genomes there was only single copy of an individual aRNH, while other genomes contained up to eleven copies of an individual aRNH. The copy number of fmRNHs per genome was also relatively low, varying from one to seven (Table 1), suggesting that due to its ubiquity and low copy number, fmRNH is the most likely candidate for the cellular RNH gene in oomycetes. However, the functions and origins of the individual aRNHs in oomycetes remain elusive.

**Table 1:** Diversity, distribution, and the number of aRNH and fmRNH domains in the studied oomycete species

Taxonomic position according to the NCBI taxonomy				RT-associated RNHs number				Individual RNHs number			
Order	Family	Genus	Species	Chronos	Archon	L1	Utopia	aRNH			fmRNH
								aRNH 1	aRNH 2	aRNH 3	
Albuginales	Albuginaceae	*Albugo*	*A. candida*	-	-	-	-	-	-	-	2
			*A. laibachii*	-	-	-	-	-	-	-	1
Peronosporales	Peronosporaceae	*Hyaloperonospora*	*H. arabidopsidis* Emoy2	57	-	88	-	-	1	-	1
		*Phytophthora*	*P. alni*	106	-	3	2	4	-	-	7
			*P. capsici*	64	-	14	16	1	1	-	1
			*P. cinnamomi var cinnamomi*	92	-	3	5	-	1	-	1
			*P. infestans* T30-4	1555	-	43	25	-	1	-	2
			*P. kernovia* 00238/432	3	-	-	1	-	1	-	1
			*P. lateralis* MPF4	30	-	7	5	-	1	-	2
			*P. parasitica* P1569	11	-	-	1	1	1	-	4
			*P. pinifolia* CBS 122922	75	-	26	2	8	2	-	2
			*P. ramorum*	120	-	11	14	-	1	-	3
			*P. sojae*	271	-	35	31	2	1	-	4
			Average	233	-	18	10	3	1	-	3
		*Phytopythium*	*P. vexans*	1	-	-	1	-	1	-	2
		*Pseudoperonospora*	*P. cubensis* MSU-1	-	-	-	-	-	-	-	1
Pythiales	Pythiaceae	*Pythium*	*P. aphanidermatum*	5	-	3	-	-	1	-	1
			*P. arrhenomanes*	5	-	5	2	-	1	-	1
			*P. insidiosum*	33	-	43	2	-	1	-	1
			*P. irregulare*	1	-	-	1	-	1	-	2
			*P. iwayamai*	1	-	3	-	-	1	-	2
			*P. ultimum var. ultimum*	-	-	74	7	-	1	-	2
			Average	9	-	26	3	-	1	-	2
Saprolegniales	Saprolegniaceae	*Aphanomyces*	*A. astaci* APO3.2	1	-	-	-	-	-	3	1
			*A. invadans* 9901.2	-	-	-	-	-	-	-	1
			Average	1	-	-	-	-	-	3	1
		*Saprolegnia*	*S. diclina* VS20	-	3	-	1	-	1	10	1
			*S. parasitica* CBS 223.65	-	2	-	-	-	1	4	1
			Average	-	3	-	1	-	1	7	1

**Fig. 2:**
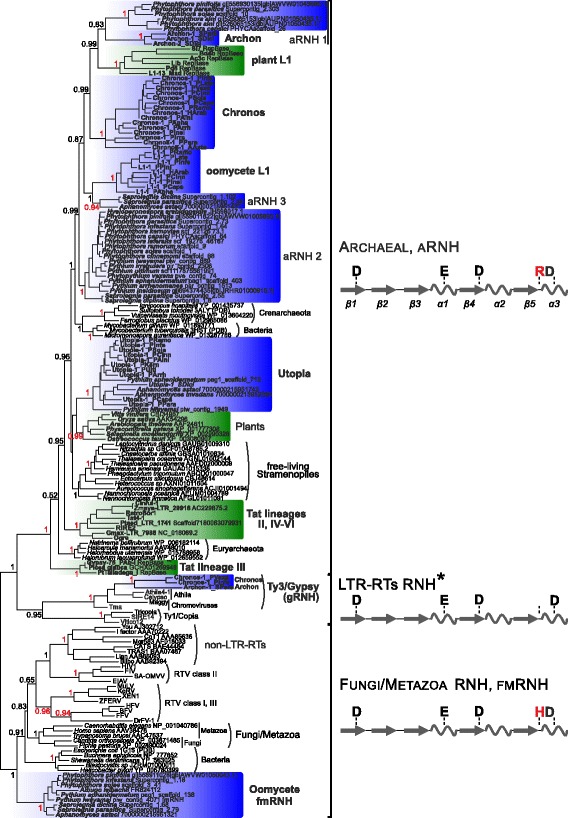
Maximum-likelihood representative tree based on the amino acid sequences of different types of type I RNHs. Approximate likelihood-ratio test (aLRT) statistical support values (unit fractions) are shown at the corresponding nodes of the tree; the values are highlighted in red if the corresponding node was additionally supported by more than 60 of 100 bootstrap replicates. Comparison of Maximum-likelihood and Bayesian reconstructions and bootstrap values are presented in Additional file 4: Figure S3. RNH lineages specific for oomycetes and plants are highlighted in blue and green gradient blocks, respectively. RTV – retroviruses. The names of oomycete non-LTR-RT and LTR-RT RNH sequences identified in the present study correspond to those in Additional file 1: Table S2. Names of RNHs of other LTR-RTs and non-LTR-RTs correspond to those in GyDB [39] and Repbase Update [21], respectively. NCBI accession numbers are indicated to the right of other RNH sequences. Schemes of the secondary structures of three subtypes of RNH with the corresponding active site residues are shown at the right of the tree. The α-helices are depicted as helices, and the β-sheets are shown as arrows. The conserved R/H residue of the active site, which varies between different RNH subtypes, is highlighted in red. *The D-E-D-D catalytic residues are not conserved in the gRNHs of Archon, Chronos and Tat LTR-RTs

**Fig. 3:**
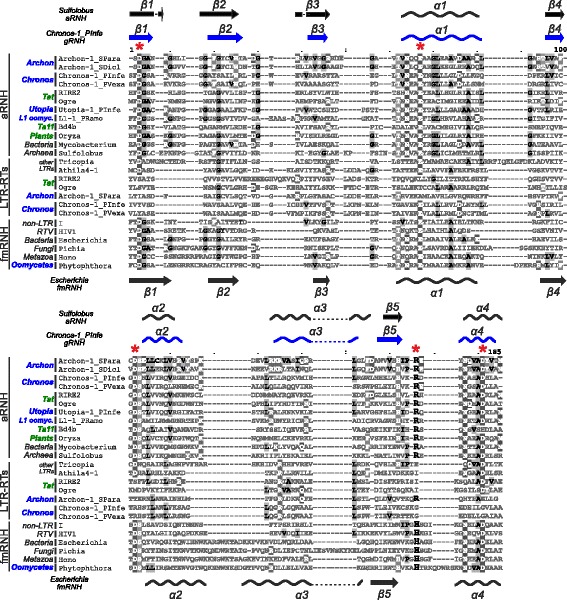
Multiple amino acid sequence alignment of different types of RNHs. The names of RNH sequences corresponding to oomycete and plant lineages are emphasized in bold and highlighted in blue and green, respectively. Archaeal RNHs, Fungi/Metazoa RNHs, and original RNHs of LTR retrotransposons are designated as aRNH, fmRNH, and LTR-RTs, respectively. Apart from RIRE2 and Ogre gRNH that were retrieved from GyDB, all the sequences are available in the Additional file 8. Conserved catalytic residues (D-E-D-R/H-D) are indicated by asterisks at the top of the alignment. The semiconservative (R/H)-residue varying between the aRNH and fmRNH is additionally denoted by the bigger font at position 166 of the alignment. The conserved residues are highlighted in shades of gray. The secondary structure of *Escherichia coli* fmRNH (PDB: 1g15_A) is shown at the bottom of the alignment. The secondary structures of oomycete Chronos-1_PInfe LTR gRNH (predicted, this study) and *Sulfolobus tokodaii* aRNH (PDB: 3aly_A) are shown at the top of the alignment. The α-helices are depicted as helices, and the β-sheets are shown as arrows

To unveil the origin of both RT-associated aRNHs and individual aRNHs in oomycetes we performed a comparative analysis of RNH genes and domains from various sources (Figs. 2 and 3, Additional file 4: Figure S3, Table 1). L1, Archon, Chronos, and Utopia oomycete aRNH domains and aRNHs of plant retrotransposons form distinct clades on the tree (Fig. 2). The identified individual aRNHs were split into three clades on the tree: aRNH 1, aRNH 2, and aRNH 3. Two clades, aRNH 1 and aRNH 3, clustered together with the aRNH domains from oomycete retrotransposons Archon and L1, respectively, although this clustering was not supported by the bootstrap. aRNH 2 formed a distinct clade that did not show any significant clustering with any RT-associated aRNHs (Fig. 2, Additional file 4: Figure S3). Notably, multiple copies of both aRNH 1 and aRNH 3 were detected in the studied oomycete genomes (Table 1). Thus, together with the potential relationship between the two aRNH groups and the RT-associated aRNHs of oomycetes, these results may suggest that aRNH 1 and aRNH 3 may represent remnants of Archon and L1 retrotransposons. In contrast, aRNH 2 was not related to RT-associated aRNHs (Fig. 2, Additional file 4: Figure S3). Therefore, it is likely that aRNH 2, in addition to fmRNH, could be a cellular RNH gene in oomycetes. This finding is also supported by the wide distribution and low copy number of aRNH 2 (Table 1).

To shed more light on the evolution of both aRNH and fmRNH in oomycetes, we mined aRNH and fmRNH homologs from the free-living Stramenopiles taxa, the closest relatives of oomycetes available in databases (Additional file 1: Table S3) using a tBLASTn search against NCBI WGS and TSA databases with oomycete aRNH and fmRNH amino acid domain sequences as queries (Fig. 2, Additional file 4: Figure S3) [25]. The results revealed aRNHs in the Stramenopiles genomes but did not detect fmRNHs (Additional file 1: Table S3). The aRNH domains of free-living Stramenopiles form a monophyletic clade on the Maximum-likelihood RNH tree (only weakly supported by the bootstrap) and a paraphyletic clade on the Bayesian tree. In addition, these RNH sequences did not show any significant clustering with other studied aRNHs (Additional file 4: Figure S3).

## Discussion

### Potential origin of aRNH and fmRNH in oomycetes

While searching for homologs of aRNH and fmRNH in oomycete genomes, we identified aRNH in both free-living Stramenopiles and oomycete taxa, while fmRNH was detected only in oomycetes (Table 1). In addition, aRNH is absent in some groups of oomycetes, likely reflecting its loss in small genome parasitic lineages, such as Albuginales [26]. One possibility is that aRNH was present in the ancestor of the Stramenopiles lineage and was vertically transmitted to oomycetes. Alternatively, aRNH might have been horizontally transferred from green plants, onto which most of the oomycete taxa examined in the present study typically parasitize [25, 27, 28]. The lack of aRNH in some oomycete genomes can be explained by the redundancy of aRNH and fmRNH functions.

The lack of fmRNH in the free-living Stramenopiles most likely indicates that oomycetes acquired this gene after the divergence from the Stramenopiles stem. The horizontal transfer of genes from fungi to oomycetes as an adaptation to parasitism on algae and plants has been previously proposed [27, 28]. Fungal genomes encode fmRNHs, which are responsible for the precise removal of RNA primers of Okazaki fragments during DNA replication and are critical for the maintenance of genome integrity (Fig. 2, Additional file 4: Figure S3) [29, 30]. Thus, it could be hypothesized that oomycetes might have acquired fmRNH through horizontal transfer together with other genes from ancient fungal lineages. However, in our phylogenetic reconstruction oomycete fmRNHs are only distantly related to fungal fmRNHs, which contradicts this hypothesis (Additional file 4: Figure S3).

### Convergence between oomycete and plant retrotransposons

In the present study we showed that based on RT phylogeny, the identified aRNH-containing oomycete L1 non-LTR-RTs, and Chronos and Archon LTR-RTs are only distantly related to the previously described aRNH-containing Ta11 L1 non-LTR-RTs and Tat LTR-RTs of green plants (Fig. 1, Additional file 2: Figure S1, and Additional file 3: Figure S2). The distinct phylogenetic positions of the elements contradict the possibility of a single origin of all aRNH-containing LTR and non-LTR retroelement from plants and oomycetes. We therefore suggest that presence of aRNH in Tat, Chronos, and Archon LTR-RTs and Ta11 L1 and oomycete L1 non-LTR-RTs could be the best explained by series of independent aRNH acquisitions by ancestors of these elements, reflecting their convergent evolution to the similar structural compositions. However, the single origin of all aRNH-containing LTR and non-LTR retrotransposons from plants and oomycetes could not be completely rejected by the phylogenetic reconstructions due to the low bootstrap support values (in contrast to the aLRT and Bayesian posterior probabilities supports) that we obtained for the paraphyletic origin of the aRNH-containing retrotransposons (Fig. 1, Additional file 2: Figure S1, and Additional file 3: Figure S2), leaving the alternative to convergent evolution still open for discussion.

The repeated sequestration and fixation of some functional domains during the evolution by diverse members of a certain genetic lineage may reflect a beneficial effect on the selection in the environment that this lineage inhabits. Previously, we proposed that the ‘dual’ RNH domains of plant Tat LTR-RTs reflected convergent evolution with vertebrate retroviruses [5]. With the discovery of Chronos and Archon LTR-RTs in oomycetes, ‘dual’ RNH acquisition may indicate a more general evolutionary tendency in all LTR-RTs. Indeed, the loss of the conserved catalytic residues (D-E-D-R/H-D) in the original Ty3/Gypsy RNH domain and their complete set in aRNH of Chronos and Archon representatives (Fig. 3) is similar to what was shown for Tat LTR-RTs [5], and resembles transformation of the original retroviral RNH to the connection (tether) RNH domain after the acquisition of new eukaryotic fmRNH in retroviruses [16] that is supported by the structural study of Ty3 reverse transcriptase [31]. Intriguingly, this evolutionary pathway may resemble an early stage in the transition of a Ty3/Gypsy retrotransposon into a retrovirus, preceding the acquisition of the infection-mediating envelope domain.

The beneficial effect from the RNH acquisition for non-LTR-RTs, however, is still poorly understood, as these elements typically rely on the host-encoded RNH activity. Furthermore, RNH could also be lost within some non-LTR-RT groups [32]. The finding of multiple examples of RNH acquisition in non-LTR-RTs therefore remains enigmatic.

The structural analysis of Chronos LTR-RTs revealed that apart from the aRNH domain, these elements also harbor CHD on the C-terminal end of the ORF2 next to the INT domain (INT-CHD), similar to the Ty3/Gypsy Chromoviruses from plants, fungi, and vertebrates [7, 9, 18, 19, 33]. Based on RT phylogeny, we showed that Chronos LTR-RTs and Chromoviruses are evolutionarily distinct from each other, thereby suggesting the convergent acquisition of the CHD by both groups. Interestingly, apart from Chromoviruses and Chronos LTR-RTs the INT-CHD domain was also reported for phylogenetically distant Ty1/Copia CoDi-I elements observed in the free-living Stramenopiles, pennate diatom, *Phaeodactylum tricornutum* [17]. See Additional file 5: Figure S4 for the multiple sequence alignment of CHDs from Chronos, Chromoviruses, and CoDI-I LTR-RTs. CHDs are widespread domains involved in chromatin remodeling in eukaryotes [34, 35]. The fusion of the CHD to the INT in LTR-RTs likely targets retrotransposon integration to the heterochromatin away from gene-rich regions [36]. Thus, multiple acquisitions of the CHD reflect the evolutionary tendency in LTR-RTs to minimize the damage to the host, while “quietly hitchhiking” its cellular machinery for retrotransposon propagation within the genome.

## Conclusions

The current understanding of the diversity of retrotransposons and other mobile elements increases with an increasing number of sequenced genomes from a broad taxa range. In the present study, we identified and characterized several groups of retrotransposons from oomycete genomes, which to our knowledge has not previously been described. Importantly, the similar patterns of acquisitions of aRNH and CHD by unrelated retrotransposon groups from oomycetes and plants suggest that these events may represent a major evolutionary trend in retroelement evolution. This trend is likely independent of the retrotransposon host genome and may reflect similarities in the fundamental organization of retrotransposon life cycle, suggesting a beneficial role for the acquired domains in this cycle.

## Methods

### Computational mining for aRNH-containing repeats in Repbase update

The complete database of prototypic repetitive sequences Repbase Update (RU, v. 20.08) [21] was downloaded and analyzed for the presence of aRNH-containing repeats. Based on a hidden Markov model profile (HMM profile), aRNH domains were mapped using hmmsearch tool of the HMMER package [37] in translations of the retrieved RU sequences. The HMM profile was constructed from the amino acid alignment of aRNH sequences from the Ustyantsev et al. [5]. Repeats without the predicted similarity to aRNH were filtered out. The remained RU repeats were initially grouped according to the taxon of origin and subsequently grouped according to repeat type.

### Computational mining for aRNH-containing retrotransposons, individual aRNH and fmRNH domains in oomycete genomes

The oomycete genomic sequences used in the present study were retrieved from public databases, as listed in Additional file 1: Table S2. To identify all retrotransposons harboring aRNH, the following algorithm was implemented using the UGENE workflow designer [38]. First, based on the aRNH HMM profile, aRNH domains were mapped using the hmmsearch tool of the HMMER [37] package in translations of the genomic DNA sequences. Second, sequences surrounding the regions of significant similarity to the aRNH profile were expanded, when possible, to 10,000 bp in both directions. Third, the enlarged sequences were screened for the presence of significant similarity to RT domains of non-LTR-RTs and LTR-RTs HMM profiles using hmmsearch. The non-LTR-RTs HMM profile was generated from the RT alignment of Repbase [21] non-LTR-RTs amino acid sequences available in the RTclass1 [12] server output. The corresponding HMM profile for LTR-RTs was constructed from the RT alignment of LTR-RTs amino acid sequences available in Gypsy Database [39]. Fourth, RT-positive sequences were divided into two groups corresponding to either non-LTR-RTs or LTR-RTs, and RT-negative sequences were filtered out, and identified aRNH sequences were retained for a further separate analysis as individual aRNHs. For each dataset, representative sequences were retrieved, and the number of elements belonging to each group (Ty3/Gypsy, L1, and Utopia) was counted by repeated BLAST [40], using ORF2 amino acid sequences of the previously identified RU aRNH-containing retrotransposons of oomycetes (Gypsy_18_PIT_I Ty3/Gypsy LTR-RT, L1-5_PI L1 non-LTR-RT, and R2I-1_PI Utopia non-LTR-RT) as seeding quires in the tBLASTn search.

Fungi/Metazoa RNHs (fmRNH) were mined using the HMM profile reconstructed based on the alignment of fmRNH amino acid sequences from Ustyantsev et al. [5] with hmmsearch, and the flanking sequences were expanded 1,000 bp in both directions.

### Characterization of the structural composition of aRNH-containing retrotransposons

For each of the identified representative retrotransposons, a detailed analysis of the structural composition was performed. We used NCBI ORFfinder [41] to identify ORFs and NCBI CD-search [42] and HHpred [43] for a subsequent homology-based mining of conserved retrotransposon-specific domains. For LTR-RT representatives, when possible, the sequences of their LTRs were predicted by aligning 5′ upstream and 3′ downstream sequences flanking ORF1 and ORF2 using BLAST [40]. Secondary structure prediction for Chronos-1_PInfe aRNH was performed using Quick2D from the MPI bioinformatics toolkit [44].

### Comparative and phylogenetic analysis

The RT amino acid sequences of the LTR-RT and non-LTR-RT representatives were aligned using hmmalign tool from the HMMER package to the corresponding HMM profiles [37]. The amino acid sequences of RNH are less conservative than RT, and a profile multiple alignment with the predicted local structures and 3D constraints (PROMALS3D) server was used to produce the alignment [45]. The alignments (refer to Additional files 6, 7, and 8 for corresponding LTR-RTs RT, non-LTR-RTs RT, and RNH alignments) were manually curated, and the phylogenetic trees were reconstructed using the maximum-likelihood and Bayesian algorithms implemented in the PhyML [46] and MrBayes [47] program tools. The best model for phylogenetic reconstruction, LG + G, was suggested using the ProtTest stand-alone tool [48] based on the Akaike Information Criterion (AIC) and Bayesian Information Criterion (BIC) for each of the alignments. In PhyML, an optimal tree topology was searched among 100 random starting trees under the subtree pruning and regrafting (SPR) algorithm, from which the tree with the largest log-likelihood value was taken, and its robustness was estimated using a Bayesian-like transformation of approximate likelihood-ratio test (aLRT, aBayes) and 100 bootstrap replicates [49]. In MrBayes, 10 split Markov chain Monte Carlo (MCMC) chains were run for 2,500,000 generations with sampling each 250 generations and discarding the first 5000 samples prior to consensus tree estimation.

## Additional files

Additional file 1: Table S1. Diversity and distribution of aRNH-containing repetitive elements identified in the Repbase Update v. 20.08 (08-30-2015) database [21]. **Table S2.** Diversity, distribution and selected representatives of identified aRNH-containing retrotransposons in the studied oomycete genomes. **Table S3.** Individual aRNHs identified in the free-living Stramenopiles species. (XLSX 40 kb)
Additional file 2: Figure S1. The complete Maximum-likelihood and Bayesian phylogenetic trees reconstructed based on the amino acid sequences of RT domain of LTR-RTs (see Additional file 6 for the alignment). Statistical support was evaluated using aBayes aLRT (unit fractions) and 100 bootstrap replicates (% after a slash), and MCMC runs (%) in Maximum-likelihood and Bayesian reconstructions, respectively, and are shown at the corresponding nodes of the tree. Bootstrap values are shown only for the main indicated clusters. Chromodomain-containing clade names are underlined, and the names of the aRNH-containing clades are indicated in blue and green for plant and oomycete LTR-RTs, respectively. The names of the oomycete LTR-RT sequences identified in the present study correspond to those in Additional file 1: Table S2. Unless otherwise stated, the names of other LTR-RTs correspond to those in GyDB [39]. (PDF 779 kb)
Additional file 3: Figure S2. The complete Maximum-likelihood and Bayesian phylogenetic trees reconstructed based on the amino acid sequences of RT domain of non-LTR-RTs (see Additional file 7 for the alignment). Statistical support was evaluated using aBayes aLRT (unit fractions) and 100 bootstrap replicates (% after a slash), and MCMC runs (%) in Maximum-likelihood and Bayesian reconstructions, respectively, and the results are shown at the corresponding nodes of the tree. Bootstrap values are shown only for the main indicated clusters. The names of the aRNH-containing clades are indicated in blue and green for plant and oomycete non-LTR-RTs, respectively. The names of oomycete non-LTR-RT sequences identified in the present study correspond to those in Additional file 1: Table S2. The names of other non-LTR-RTs correspond to those in Repbase Update [21]. (PDF 366 kb)
Additional file 4: Figure S3. The complete Maximum-likelihood and Bayesian trees reconstructed based on different type I RNH amino acid sequences (see Additional file 8 for the alignment). Statistical support was evaluated using aBayes aLRT (unit fractions) and 100 bootstrap replicates (% after a slash), and MCMC runs (%) in Maximum-likelihood and Bayesian reconstructions, respectively, and the results are shown at the corresponding nodes of the tree. Bootstrap values are shown only for the main indicated clusters. The names of the RNH clades from plant and oomycete genomes are highlighted in green and blue, respectively. The names of oomycete non-LTR-RT and LTR-RT RNH sequences identified in the present study correspond to those in Additional file 1: Table S2. Names of RNHs of other LTR-RTs and non-LTR-RTs correspond to those in GyDB [39] and Repbase Update [21], respectively. NCBI accession numbers are indicated to the right of other RNH sequences. (PDF 863 kb)
Additional file 5: Figure S4. Multiple amino acid sequence alignment of CHDs from LTR-RTs and human Chromodomain Protein Y-Like 2 (PDB accession number 5JJZ_A). Additional information about the amino acid conservation is shown as a sequence Logo generated from the alignment, which is positioned at the bottom. (PDF 1096 kb)
Additional file 6: Multiple amino acid sequence alignment of RT domains from diverse LTR-RTs constructed and used for the phylogenetic reconstruction in the present study. (TXT 58 kb)
Additional file 7: Multiple amino acid sequence alignment of RT domains from diverse non-LTR-RTs constructed and used for the phylogenetic reconstruction in the present study. (TXT 83 kb)
Additional file 8: Multiple amino acid sequence alignment of RNH genes and domains from diverse taxa constructed and used for the phylogenetic reconstruction in the present study. (TXT 45 kb)

## Abbreviations

aRNH: RNH of archaeal and plant origin
CHD: Chromodomain
fmRNH: RNH of Fungi/Metazoa origin
INT: Integrase
LTR-RTs: Long terminal repeat retrotransposons
non-LTR-RTs: Non-long terminal repeat retrotransposons
ORF: Open reading frame
RLE: Restriction-like endonuclease
RNH: Ribonuclease H
RT: Reverse transcriptase
RU: Repbase update database
