# Supplementary material for: Convergence of retrotransposons in oomycetes and plants
Source: Mob DNA. 2017 Mar 14;8:4. doi: 10.1186/s13100-017-0087-y (PMC5348765; doi:10.1186/s13100-017-0087-y)
Supplement: Additional file 2: Figure S1. — The complete Maximum-likelihood and Bayesian phylogenetic trees reconstructed based on the amino acid sequences of RT domain of LTR-RTs (see Additional file 6 for the alignment). Statistical support was evaluated using aBayes aLRT (unit fractions) and 100 bootstrap replicates (% after a slash), and MCMC runs (%) in Maximum-likelihood and Bayesian reconstructions, respectively, and are shown at the corresponding nodes of the tree. Bootstrap values are shown only for the main indicated clusters. Chromodomain-containing clade names are underlined, and the names of the aRNH-containing clades are indicated in blue and green for plant and oomycete LTR-RTs, respectively. The names of the oomycete LTR-RT sequences identified in the present study correspond to those in Additional file 1: Table S2. Unless otherwise stated, the names of other LTR-RTs correspond to those in GyDB [39]. (PDF 779 kb) [file 13100_2017_87_MOESM2_ESM.pdf]

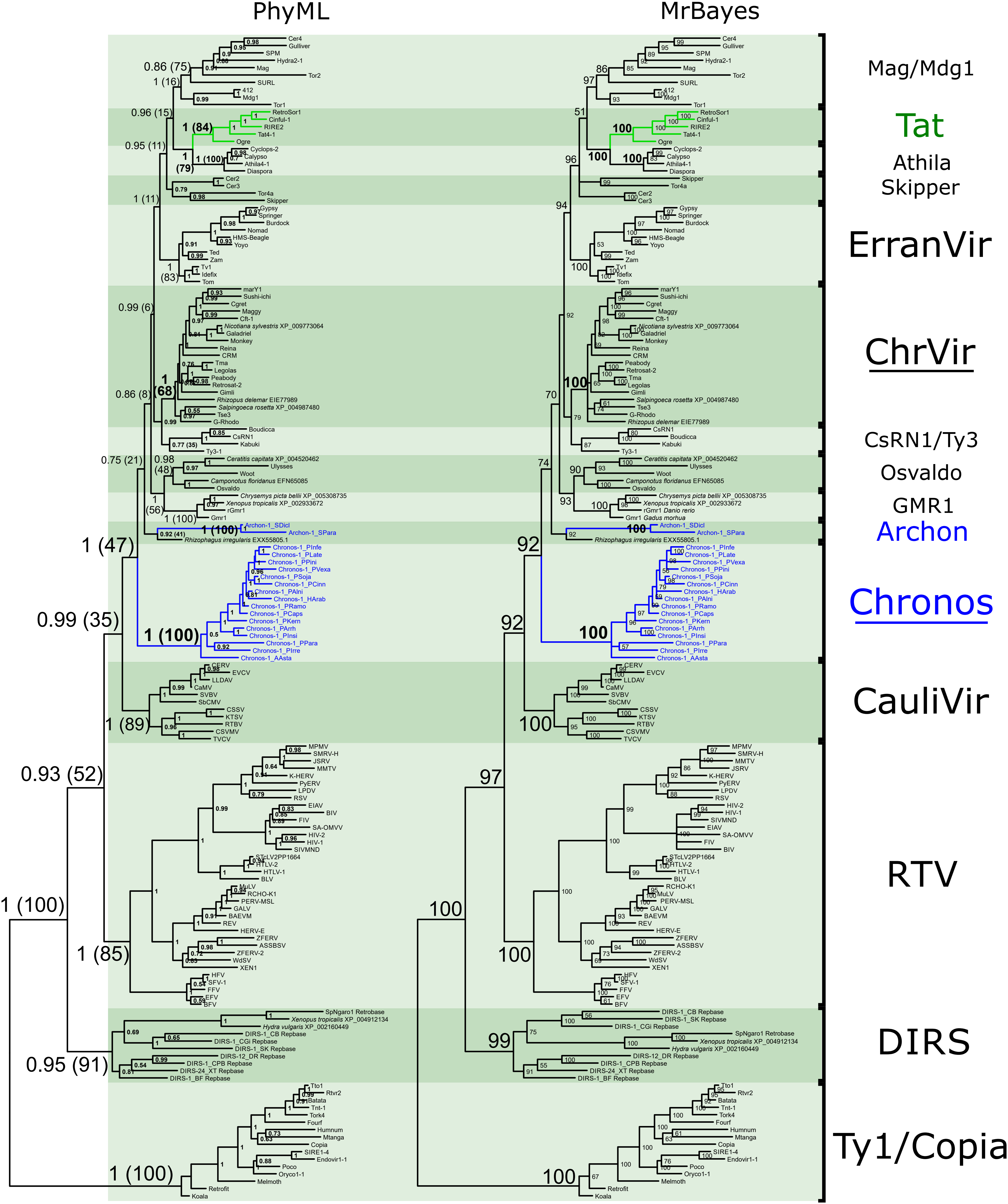

PhyML
MrBayes
Mag/Mdg1
Tat
Athila
Skipper
ErranVir
ChrVir
CsRN1/Ty3
Osvaldo
GMR1
Archon
Chronos
CauliVir
RTV
DIRS
Ty1/Copia
Cer4
Gulliver
SPM
Hydra2-1
Mag
Tor2
SURL
412
Mdg1
Tor1
RetroSor1
Cinful-1
RIRE2
Tat4-1
Ogre
Cyclops-2
Calypso
Athila4-1
Diaspora
Cer2
Cer3
Tor4a
Skipper
Gypsy
Springer
Burdock
Nomad
HMS-Beagle
Yoyo
Ted
Zam
Tv1
Idefix
Tom
marY1
Sushi-ichi
Cgret
Maggy
Cft-1
Nicotiana sylvestris XP_009773064
Galadriel
Monkey
Reina
CRM
Tma
Legolas
Peabody
Retrosat-2
Gimli
Rhizopus delemar EIE77989
Salpingoeca rosetta XP_004987480
Tse3
G-Rhodo
Boudicca
CsRN1
Kabuki
Ty3-1
Ceratitis capitata XP_004520462
Ulysses
Woot
Camponotus floridanus EFN65085
Osvaldo
Chrysemys picta bellii XP_005308735
Xenopus tropicalis XP_002933672
rGmr1
Gmr1
rGmr1 Danio rerio
Gmr1 Gadus morhua
Archon-1_SDicl
Archon-1_SPara
Rhizophagus irregularis EXX55805.1
Chronos-1_PInfe
Chronos-1_PLate
Chronos-1_PPini
Chronos-1_PVexa
Chronos-1_PSoja
Chronos-1_PCinn
Chronos-1_PAlni
Chronos-1_HArab
Chronos-1_PRamo
Chronos-1_PCaps
Chronos-1_PKern
Chronos-1_PArrh
Chronos-1_PInsi
Chronos-1_PPara
Chronos-1_PIrre
Chronos-1_AAsta
CERV
EVCV
LLDAV
CaMV
SVBV
SbCMV
CSSV
KTSV
RTBV
CSVMV
TVCV
MPMV
SMRV-H
JSRV
MMTV
K-HERV
PyERV
LPDV
RSV
EIAV
BIV
FIV
SA-OMVV
HIV-2
HIV-1
SIVMND
STcLV2PP1664
HTLV-2
HTLV-1
BLV
MuLV
RCHO-K1
PERV-MSL
GALV
BAEVM
REV
HERV-E
ZFERV
ASSBSV
ZFERV-2
WdSV
XEN1
HFV
SFV-1
FFV
EFV
BFV
SpNgaro1 Retrobase
Xenopus tropicalis XP_004912134
Hydra vulgaris XP_002160449
DIRS-1_CB Repbase
DIRS-1_CGi Repbase
DIRS-1_SK Repbase
DIRS-12_DR Repbase
DIRS-1_CPB Repbase
DIRS-24_XT Repbase
DIRS-1_BF Repbase
Tto1
Rtvr2
Batata
Tnt-1
Tork4
Fourf
Humnum
Mtanga
Copia
SIRE1-4
Endovir1-1
Poco
Oryco1-1
Melmoth
Retrofit
Koala
