# Supplementary figures and images for: Convergence of retrotransposons in oomycetes and plants
Source: Mob DNA. 2017 Mar 14;8:4. doi: 10.1186/s13100-017-0087-y (PMC5348765; doi:10.1186/s13100-017-0087-y)

PhyML

MrBayes

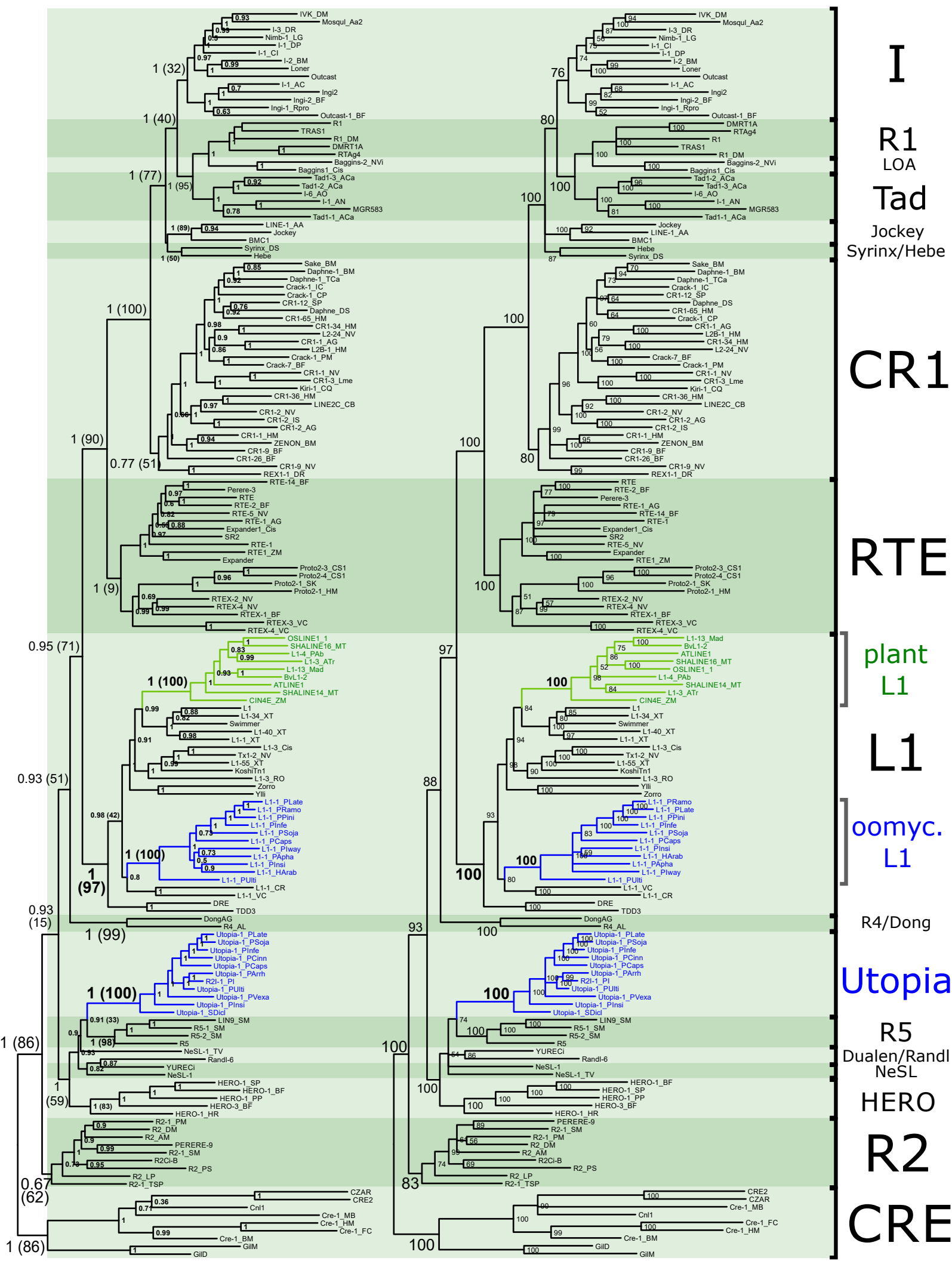

Supplement: Additional file 3: Figure S2. — The complete Maximum-likelihood and Bayesian phylogenetic trees reconstructed based on the amino acid sequences of RT domain of non-LTR-RTs (see Additional file 7 for the alignment). Statistical support was evaluated using aBayes aLRT (unit fractions) and 100 bootstrap replicates (% after a slash), and MCMC runs (%) in Maximum-likelihood and Bayesian reconstructions, respectively, and the results are shown at the corresponding nodes of the tree. Bootstrap values are shown only for the main indicated clusters. The names of the aRNH-containing clades are indicated in blue and green for plant and oomycete non-LTR-RTs, respectively. The names of oomycete non-LTR-RT sequences identified in the present study correspond to those in Additional file 1: Table S2. The names of other non-LTR-RTs correspond to those in Repbase Update [21]. (PDF 366 kb) [file 13100_2017_87_MOESM3_ESM.pdf]

# PhyML

# MrBayes

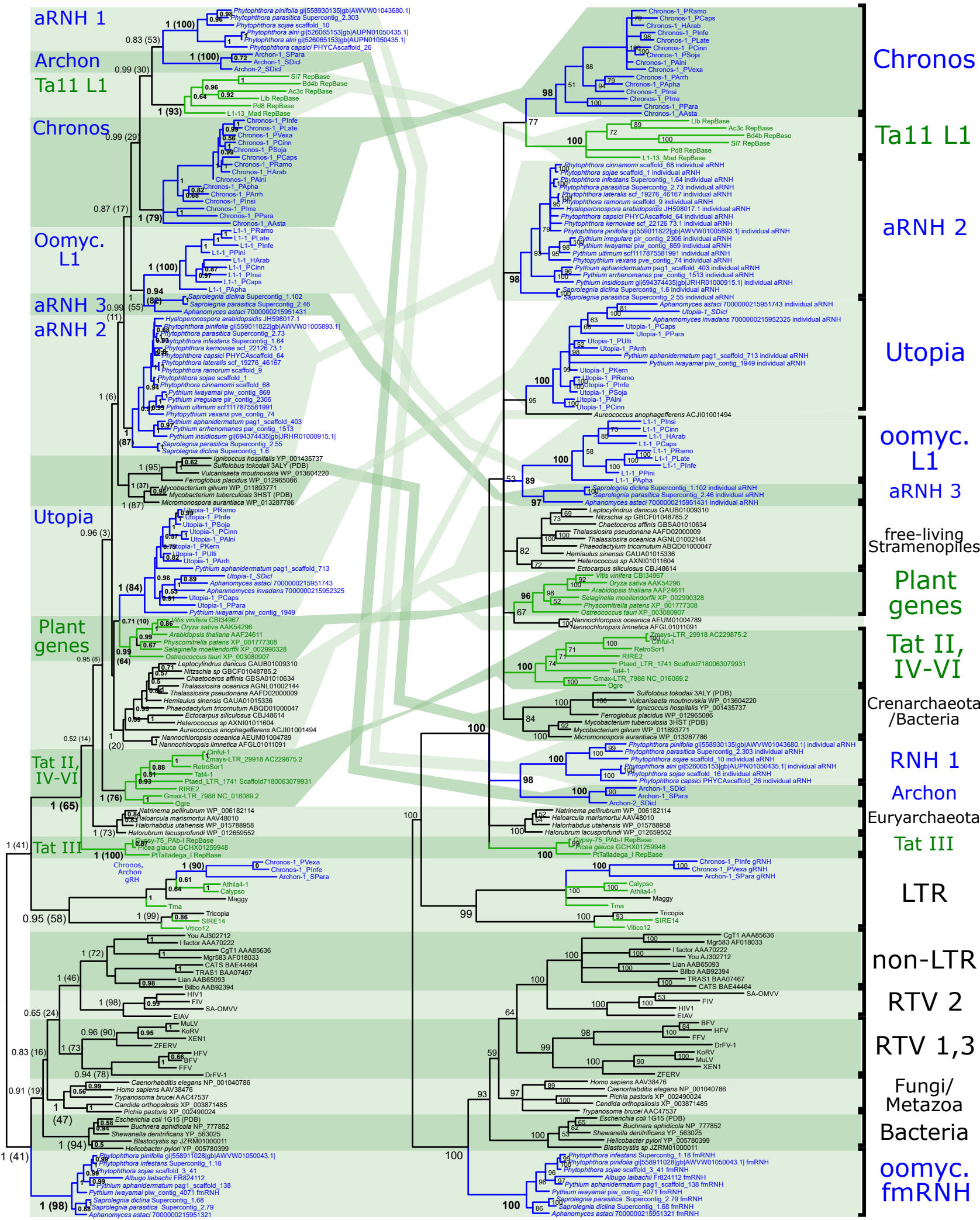

Supplement: Additional file 4: Figure S3. — The complete Maximum-likelihood and Bayesian trees reconstructed based on different type I RNH amino acid sequences (see Additional file 8 for the alignment). Statistical support was evaluated using aBayes aLRT (unit fractions) and 100 bootstrap replicates (% after a slash), and MCMC runs (%) in Maximum-likelihood and Bayesian reconstructions, respectively, and the results are shown at the corresponding nodes of the tree. Bootstrap values are shown only for the main indicated clusters. The names of the RNH clades from plant and oomycete genomes are highlighted in green and blue, respectively. The names of oomycete non-LTR-RT and LTR-RT RNH sequences identified in the present study correspond to those in Additional file 1: Table S2. Names of RNHs of other LTR-RTs and non-LTR-RTs correspond to those in GyDB [39] and Repbase Update [21], respectively. NCBI accession numbers are indicated to the right of other RNH sequences. (PDF 863 kb) [file 13100_2017_87_MOESM4_ESM.pdf]

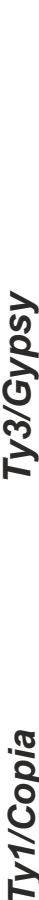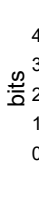

Supplement: Additional file 5: Figure S4. — Multiple amino acid sequence alignment of CHDs from LTR-RTs and human Chromodomain Protein Y-Like 2 (PDB accession number 5JJZ_A). Additional information about the amino acid conservation is shown as a sequence Logo generated from the alignment, which is positioned at the bottom. (PDF 1096 kb) [file 13100_2017_87_MOESM5_ESM.pdf]
